# Supplementary material for: Predictors of radiological aggravations of pulmonary MAC disease
Source: PLoS One. 2020 Aug 6;15(8):e0237071. doi: 10.1371/journal.pone.0237071 (PMC7410298; doi:10.1371/journal.pone.0237071)
Supplement: Attachment — Submitted filename: Response to Reviewers.docx [file pone.0237071.s001.docx]

**PONE-D-19-29751
Predictors of exacerbations of pulmonary MAC disease
PLOS ONE


Academic Editor**
We wish to thank the academic editor for the comments.

**Comment**

1) Please provide additional details regarding participant consent. In the ethics statement in the Methods and online submission information, please ensure that you have specified (1) whether consent was informed and (2) what type you obtained (for instance, written or verbal). If your study included minors, state whether you obtained consent from parents or guardians. If the need for consent was waived by the ethics committee, please include this information.

**Response**

We added the following on Page 5, Lines 18-20: “The need for written informed patient consent was waived in view of the retrospective and observational nature of the study.”

Details about inclusion in MAC statistics are on the website for Toho University Medical Center Ohashi Hospital, and we obtained verbal informed consent for inclusion. The need for written informed patient consent was waived in view of the retrospective and observational nature of the study. As our study did not include minors, we did not obtain consent from parents or guardians.

**Comment**

Please also clarify whether the 'Special Committee of Toho University Ohashi Medical Center' is an ethics committee that reviews research on human subjects.

**Response**

We added the following on Page 5, Lines 22-23: “This committee is an ethics board that reviews research on human subjects.”

**Comment**

Your ethics statement must appear in the Methods section of your manuscript. If your ethics statement is written in any section besides the Methods, please move it to the Methods section and delete it from any other section. Please also ensure that your ethics statement is included in your manuscript, as the ethics section of your online submission will not be published alongside your manuscript.

**Response**

As you suggested, we moved the ethics statement to the Methods section and deleted it from the other section.

**Comment
Reviewer #1:**

The data presented in the paper looks interesting but not novel. It is too preliminary to be accepted as a research article in your esteemed journal. It could be evaluated for a short note or communication after following changes are made.
Many of the parameter shows very high standard deviation. It would be more appropriate to give the interquartile range for all the parameters to understand the significance better. The conclusions are questionable in the current format.
The manuscript requires major revision in the written English language and cannot be accepted in the present format.

**Response**

We wish to thank the reviewer for the comments.

The new information provided by this study is that the most critical exacerbating factor of pulmonary MAC disease over 1 year is the presence of extensive abnormal shadows, especially the presence of abnormal shadows in ≥3 lobes in the lung. This information had never been reported by previous studies.

Our scoring system involves simply counting the number of abnormal lesions in the lung fields divided into six zones based on anatomical structures; thus, this system is far removed from previous detailed scoring systems, which are complicated and require much more effort. In contrast, our scoring system is simple and quick, so it can be easily performed in actual clinical practice.

As you suggested, we changed the date to interquartile ranges for all parameters to understand the significance better.

We also asked a native English-speaking medical editor to check the paper.

**Comment
Reviewer #2:**

This is a retrospective study looking into factors associated with the exacerbation of pulmonary MAC infection. The statistical analysis mainly focused on the association between sex and smokers, as shown in Tables 2, 3 and 4. However, these analyses are indeed redundant and could be simplified into one table in Table 5 that looks into the main theme of this study, which is to identify the factors associated with the exacerbation of pulmonary MAC disease. An assumption could be made that the authors do not know the statistical method and taking such a redundant way. Besides, the multivariate analysis is not clear of what the authors are looking for, and the criteria for selecting the variates seem to be not justified making the results unreliable. Not only the statistical analyses are
unreliable, but the results also are not new and tell the readers nothing additional to the clinical practice.

**Response**

We wish to thank the reviewer for the comments.

The number of patients with pulmonary MAC disease is increasing worldwide, especially among women and never-smokers. The roles of female hormones and adiponectin have been suggested, though the underlying mechanisms are yet to be determined. We did a simple analysis of this, and in the process, we found it was easier to detect MAC disease relatively early in smokers, due to the presence of other diseases. Therefore, we decided to perform additional analysis.

As you suggested, the presence of more extensive abnormal shadows has been previously shown to be associated with exacerbations of pulmonary MAC disease.

The new information provided by this study is that the most critical exacerbating factor of pulmonary MAC disease over 1 year is the presence of extensive abnormal shadows, especially the presence of abnormal shadows in ≥3 lobes in the lung. This information had never been reported by previous studies.

Our scoring system involves simply counting the number of abnormal lesions in the lung fields divided into six zones based on anatomical structures; thus, this system is far removed from previous detailed scoring systems, which are complicated and required much more effort. In contrast, our scoring system is simple and quick, so it can be easily performed in actual clinical practice. We therefore believe this paper merits publication.

Regarding statistics, some of the parameters showed high standard deviation. It would be more appropriate to give the interquartile range for all parameters to understand the significance better. Thus, we changed all parameters to include interquartile ranges.
