# Supplementary material for: Predictors of radiological aggravations of pulmonary MAC disease
Source: PLoS One. 2020 Aug 6;15(8):e0237071. doi: 10.1371/journal.pone.0237071 (PMC7410298; doi:10.1371/journal.pone.0237071)
Supplement: Attachment — Submitted filename: Responses To Reviewers.docx [file pone.0237071.s002.docx]

**PONE-D-19-29751
Predictors of exacerbations of pulmonary MAC disease
PLOS ONE


Academic Editor**
We wish to thank the academic editor for the comments.

**Comment**

1) A major issue in this manuscript that has not been satisfactorily addressed is the statistical analysis of the retrospective data. Therefore, it is important that the authors explicitly explain the statistical analysis and the tools used etc., in the methods or results section (refer reviewer comment).  Also, it is important to discuss the limitations of this study. Include a paragraph in the discussion section that describes all type of limitations applicable to this retrospective analysis.

**Response**

As you suggested, our statistical analysis of the retrospective data are difficult to understand, so we changed Table 5 as follows.

The odds ratio between the dependent factor (exacerbating factor) and the independent factor has been reduced to 1 or less.

Therefore, we changed “lung disease other than mycobacterial disease, n (%)” to “No lung disease other than MAC disease, n (%)” and corrected each numerical value in Table 5.

As mentioned, when we had consulted with experts about statistics, multivariate analysis with items that are similar was not considered statistically accurate.

Therefore, multivariate analysis was performed using radiological factor (larger affected area), comorbidity (absence of other lung disease), sex (female sex), and smoking (never-smoker), and we created Figure 2 separately.

As regards limitations, we added the following on Page 11,Line 12,

“**Limitations** Some limitations in our study should be addressed. This study is limited by retrospective nature without randomization, and single-institutional study. In addition, this study has been performed only for those with a definitive diagnosis of MAC disease. Suspected MAC disease, for example, the cases that bacteria were detected by only once with sputum , and the cases that have been treated with MAC diagnosis in the past, have been excluded. So, it is presumed that the number of cases was limited, the number of cases being treated was small, and the elderly were many.”

**Comment

Reviewer #1:**

The manuscript titled “Predictors of exacerbation of pulmonary MAC disease” is written well and interesting to note the new scoring system for pulmonary involvement. The authors claim that “The new information provided by this study is that the most critical exacerbating factor of pulmonary MAC disease over 1 year is the presence of extensive abnormal shadows, especially the presence of abnormal shadows in ≥3 lobes in the lung. This information had never been reported by previous studies”. There is a certain bias in the age group who are all elders above 69. It does not represent the age distribution within a normal population. Besides, there are no representative images to explain the scoring system. The data on the co-relation to sex and smoking status does not add any value to the idea mentioned in the title. Also, only 39 people have received MAC therapy. Although this is a very notable finding by the authors, in my opinion, it does not warrant its publication as a full-length manuscript but a brief communication.

**Response**

We wish to thank the reviewer for the comments.

This study involved only those with a definitive diagnosis of MAC disease. Suspected cases of MAC disease, for example, the cases in which bacteria were detected only once in sputum and cases treated with a MAC diagnosis in the past, were excluded. Thus, it is presumed that the number of cases was limited, the number of cases being treated was small, and there were many elderly patients.

As you suggested, we added the following on Page11, Lines12, “**Limitations** Some limitations in our study should be addressed. This study is limited by retrospective nature without randomization, and single-institutional study. In addition, this study has been performed only for those with a definitive diagnosis of MAC disease. Suspected MAC disease, for example, the cases that bacteria were detected by only once with sputum and so on, the cases that have been treated with MAC diagnosis, have been excluded. So, it is presumed that the number of cases was limited, the number of cases being treated was small, and the elderly were many.”

As regards our scoring system, our scoring system involves just counting the number of abnormal lesions in the lung fields divided into six zones based on anatomical structures. Thus, we did not show the scoring system.

As mentioned, we deleted Table 5 that was not needed.

However, the number of patients with pulmonary MAC disease is increasing worldwide, especially among women and never-smokers. We did a simple analysis of it, and in the process, we noted that is easier to detect MAC disease relatively early in smokers, because of other diseases. Therefore, we decided to perform an additional analysis.

**Comment

Reviewer #2:**

I appreciate your response to my comments. Statistical methods are basically the same. As previously mentioned, there is nothing novel to our clinical practice. The author's comment indicates the presence of a scoring system, which is not mentioned in the manuscript.

**Response**

We wish to thank the reviewer for the comments.

As mentioned, our statistical analysis of the retrospective data was difficult to understand, so we changed Table 5 as follows. The odds ratio between the dependent factor (exacerbating factor) and the independent factor was reduced to 1 or less. Therefore, we changed “lung disease other than mycobacterial disease, n (%)” to “No lung disease other than MAC disease, n (%)” and corrected each numerical value in Table 5.

As mentioned, when we had consulted with experts about statistics, multivariate analysis with the items that are similar was not considered statistically accurate.

Therefore, multivariate analysis was performed using radiological factor (larger affected area), comorbidity (absence of other lung disease), sex (female sex), and smoking (never smoker), and we created Figure 2 separately.

I think, the new information provided by this study is that the most critical exacerbating factor of pulmonary MAC disease over 1 year is the presence of extensive abnormal shadows, especially the presence of abnormal shadows in ≥3 lobes in the lung, and tat female sex and never-smoker were not associated with exacerbations over 1 year.
