# Supplementary material for: Predictors of radiological aggravations of pulmonary MAC disease
Source: PLoS One. 2020 Aug 6;15(8):e0237071. doi: 10.1371/journal.pone.0237071 (PMC7410298; doi:10.1371/journal.pone.0237071)
Supplement: Attachment — Submitted filename: Responses.docx [file pone.0237071.s004.docx]

**PONE-D-19-29751R2
Predictors of radiologic aggravations of pulmonary MAC disease
PLOS ONE


Academic Editor:**

We wish to thank the academic editor for the comments.

**Comment**

There are lots of issues in this manuscript including definition of "exacerbation" (refer reviewer#3 comments), limited number of samples, data interpretation and statistical analysis. As suggested by the reviewers, the authors should consider presenting the data of only treatment-naive patients in the main text. Also, summarize the limitations of this studies and discuss the discrepancy in the findings between this study and previously reported similar studies.

**Response**

As you suggested, we now present the data only of treatment-naive patients in the main text. We also replaced “exacerbation” with “radiologic aggravation” and added the following on Page 10, Line 19, “In this study, disease progression was defined as aggravation on the radiological image.”

As you pointed out, the study period was extended to December 2018 due to the small number of cases, and we also corrected the statistical analysis.

We summarized the limitations of this study and discussed the discrepancy in the findings between this study and previously reported similar studies.

**Reviewer**

We wish to thank the reviewer for the comments.

**Comment**

The authors investigated the predictors of exacerbations of pulmonary MAC lung disease. Although the topic addressed in this manuscript is interesting, some of the interpretations underlying the authors' approach to be flawed. Additional description and further clarification is needed.

Major comments.

1. The description of the results and the findings of this study were quite confusing. I think this is because the authors used two different definitions with regard to the definition of “exacerbation”. That is, “exacerbation” was defined as “the frequency of MAC therapy” in Table 2, Table 3, and Page 10 line 14-16. In contrast, “exacerbation” was defined as “radiologic aggravation” in Figure 2, Table 4, Page 9 line 1-12, and Page 10 line 12-14. Previous studies (Clinical Infectious Diseases 2017;65(6):927–34, Eur Respir J 2017; 49: 1600537, BMJ Open 2015;5:e008058, <http://dx.doi.org/10.5588/ijtld.13.0792>) adopted either, but not both, as a definition of exacerbation.

**Response**

As suggested, we deleted Table 2 and Table 3, and we present the data only of treatment-naive patients in the main text. We also replaced “exacerbation” with “radiologic aggravation” and added the following on Page 10, Line 19, “In this study, disease progression was defined as aggravation on the radiological image.”

**Comment**

2. Moreover, in contrast to the authors’ description in Page 4 line 17-21, I think there is no previous studies reporting that sex and smoking status were related to the exacerbation (the frequency of MAC therapy) of MAC lung disease. I believe that, if there is any relationship, this may be due to the higher number of fibrocavitary types in men and smokers compared with women and non-smokers. Although radiologic types were not related to the exacerbation in this study, it was likely that the number of enrolled subjects were small to reveal clinical significance. Likewise, there is no reason that sex and smoking status might be related to the radiologic deterioration of MAC lung disease. Therefore, I think that Table2,3, and its relevant descriptions in the Results and Discussion sections are unnecessary because these results had little clinical implications.

**Response**

As you suggested, there have been no previous studies showing that sex and smoking status were related to the exacerbation (the frequency of MAC therapy) of MAC lung disease.

Therefore, we changed the following on Page 4, Line 21, “In contrast to prevalence, whether sex and smoking status could be factors aggravating pulmonary MAC disease remains unknown.”

As you pointed out, the study period was extended to December 2018 due to the small number of cases, and we also corrected the statistical analysis.

**Comment**

3. In contrast to previous studies concerning the predictors of radiologic deterioration enrolled both the patients received treatment and treatment-naïve patients (Clinical Infectious Diseases 2017;65(6):927–34, BMJ Open 2015;5:e008058, <http://dx.doi.org/10.5588/ijtld.13.0792>), the authors performed their analysis of predictors of exacerbation using only the data of treatment-naïve patients. I think this is the novel finding of this study. I recommend that the authors focus solely on this part and revise the paper as a whole.

**Response**

As you suggested, we focused solely on the data of treatment-naïve patients and revised the paper as a whole.

**Comment**

4. The term “exacerbation” is confusing. Because “exacerbation” is usually referred to the clinical situation that required treatment. I think “radiologic deterioration” or “radiologic aggravation” should be used instead of “exacerbation” throughout the manuscript.

**Response**

As you suggested, we replaced “exacerbation” with “radiologic aggravation” throughout the manuscript.

**Comment**

5. Page 6, line 6: Reference is needed for this description.

**Response**

We added a reference.

**Comment**

6. The authors should add the data about the number and the percentage of patients with aggravation, no change, and improvement.

**Response**

As previously mentioned, we deleted Table 2 and Table 3.

**Comment**

7. Page 7, line 4-line 6

The analysis of radiographic findings in patients received treatment was not found in the manuscript. However, as I suggested above, I recommend authors to revise the manuscript excluding the patients underwent treatment.

**Response**

As you suggested, we revised the manuscript excluding the patients who underwent treatment.

**Comment**

8. Statistical analysis

1) For the multivariate logistic analysis, the odds ratio (OR) is used. The hazard ratio is used for the Cox regression analysis.

2) In addition, higher OR should be associated with an increased risk of radiologic deterioration. However, in Figure 2, lower OR was associated with an increased risk of radiologic deterioration.

**Response**

As you suggested, we changed “HR” to “OR” and changed it so that higher OR was associated with an increased risk of radiologic aggravation.

**Comment**

9. In Table 1, all data are expressed as median value. However, in the Results section, the authors described “average” BMI and “average” number of abnormal lung zone. The mean and median values should be clearly distinguished. Please note that variables with normal distribution should be expressed as mean +/- standard deviation.

**Response**

In the previous revision, it was pointed out that the mean ± standard deviation should be changed to the median value due to the small number of cases. As suggested, we clearly distinguished the mean and median values.

**Comment**

10. Author might want to add new Table according to the radiologic aggravation, instead of Table 4.

**Response**

As suggested, we changed “exacerbation” to “radiologic aggravation.”

**Comment**

11. In Table 4, “no” lung disease other than MAC disease was a statistically significant predictor in univariate analysis. However, in the Result and Discussion section, it was described that “fewer” lung disease other than MAC was a significant factor. I think that “no” and “fewer” was totally different.

**Response**

As suggested, we corrected it to “no lung disease other than MAC”.

**Comment**

12. In Table 4, it seems that “zone of radiologic findings”, involved lobes ≥2, and involved lobes ≥3 were variables that were highly correlated with one another. In the multivariate logistic analysis, if two or more variables are highly correlated with one another, it is hard to get good estimates of their distinct effects on some dependent variable. The authors should check multicollinearity in their statistical analysis.

**Response**

As you suggested, if two or more variables are highly correlated with one another, it is hard to obtain good estimates of their distinct effects on a dependent variable.

Therefore, regarding the number of abnormal lesions, the sensitivity and specificity of the radiologic aggravation prediction model were calculated for each score value. We added Figure 3 and Figure 4. The performance of the radiologic aggravation prediction model was evaluated using the receiver operating characteristic (ROC) curve with the calculation of the area under the ROC curve.

**Comment**

13. Page 10, line 4-line 5

Such results cannot be found in the manuscript.

**Response**

We deleted the sentence.

**Minor comments.**

1. Page 4, line 10-line 11: The authors might want to use “guideline-based therapy” instead of “standard therapy”.

**Response**

As you suggested, we used “guideline-based therapy” instead of “standard therapy”.

**Comment**

2. Page 6, line 19: clinical course 🡪 radiologic image?

**Response**

We changed “clinical course” to “radiologic image”.

**Comment**

3. Page 8, line 1: *M. avium* – 100/128 (78.1%), *M. intracellulare* – 21/128 (16.4%), mixed infection – 7/128 (5.5%)

**Response**

We corrected it.

**Comment**

4. Page 8, line 5: BMI 19.1 – However, BMI was 19.0 in Table 1

**Response**

We corrected it.

**Comment**

5. Tables: positive MAC smear 🡪 positive AFB smear

**Response**

We replaced “positive MAC smear” with “positive AFB smear,” as suggested.

**Comment**

6. Page 10, line 18, Page 11, line 1: treatment “resistance” is an inappropriate word.

**Response**

We deleted the term.
