# Supplementary material for: Predictors of radiological aggravations of pulmonary MAC disease
Source: PLoS One. 2020 Aug 6;15(8):e0237071. doi: 10.1371/journal.pone.0237071 (PMC7410298; doi:10.1371/journal.pone.0237071)
Supplement: Attachment — Submitted filename: Responses.docx [file pone.0237071.s006.docx]

**PONE-D-19-29751R3
Predictors of radiologic aggravations of pulmonary MAC disease
PLOS ONE**

**Academic Editor:**
We wish to thank the academic editor for the comments.

Our previous study period was extended to December 2018 due to the small number of cases; thus, we changed the project registration number with the ethics committee from H16053 to H20004.

**Reviewer #1:** The manuscript “Predictors of radiologic aggravations of pulmonary MAC disease” is a neat and simple analysis. However, discussion of the results obtained should be more precise and clearer to benefit the readers. Grammar and language can be redone more rigourously. Overall formatting needs to be uniform. References can be more updated ones

We wish to thank the reviewer for the comments.

**Comment**

Page 4

Line 3 : Please update the references. The quoted ones are more than a decade old.
**Response**

We updated the references (#1-#5).

**Comment**

Page 8
Lines 13-17 can be simplified for easy understanding
**Response**

We changed the following sentences.

“The results indicated that no lung diseases other than MAC, more extensive radiological findings, and positive AFB smear were significantly associated with radiologic aggravations, whereas female sex and never-smoker were not found to be significantly associated with radiologic aggravations.”

→”The univariate analysis showed that no lung diseases other than MAC, more extensive radiological findings, and positive AFB smear were significantly associated with radiological aggravations.”

**Comment**

Line 18 “Multivariate analysis was performed with items that showed significant differences on univariate analysis”. Please change the word “items” to a more scientific word.

**Response**

As you mentioned, we replaced “items” with “factors”.

**Comment**

Line 23 Please elaborate “additional analysis”
**Response**

We added “ ROC curve analysis” after additional analysis.

**Comment**

Page 9
Line 16 -18 “A recent biological study reported the role of oestrogen in the development of pulmonary MAC disease, whereas the role of sex in disease susceptibility has yet to be determined”
The quoted reference dates back to 2001. Please use an update reference or avoid the word recent.

**Response**

As you suggested, we updated the references (#22).

**Comment**

Line 18-19 “Generally, pulmonary MAC diseases develop more frequently in thin patients”. Please provide appropriate reference.
**Response**

We added the references (#23-25 ).

**Comment**

Line 20 – Nutritional status was suggested by whom. Please be more scientific in discussion.

**Response**

It was also pointed out that the nutritional status and so on, as an onset factor, has little relationship to the main findings of present study. Thus, we deleted the sentence about the onset factor appropriately.

**Comment**

Line 22 – “Adiponectin might be involved”. This could be elaborated.

**Response**

We think this is true, but, as described previously, we deleted the sentence about the onset factor.

**Comment**

Page 10, 11 When you discuss the association of positive smear as an aggravating factorm it needs to be more precise and clearer. It is better to rewrite the univariate and multivariate analysis results more clearly.

**Response**

To make the univariate and multivariate analysis results easier to understand, we added

the following on Page 8, Lines 16-17, “(no lung diseases other than MAC, more extensive radiological findings, and positive AFB smear)”

**Comment**

Table 2: Please change the “See footnotes of Tables 1 and 2 for expansions of abbreviations “ to table 1 and not as table 1 and 2. Mention interquartile ranges and significant * p value for benefit of readers.

**Response**

We replaced “Table 1 and Table 2” with “Table 1”.

We added interquartile ranges and significant * p values.

**Comment**

Figure 3: please clearly define the Y axis and explain the same in legend

**Response**

We clearly defined the Y axis and provided an explanation in the legend.

**Comment**

Figure 4: Please explain the plot and mention the cutoff rather than just giving the title
**Response**

We added “zones of abnormal findings, AUC= 0.765”in Figure 4 and the cutoff value in the Figure legend.

**Reviewer #3:** The authors have responded well to my suggestions and revised the paper superbly. However, some major corrections and further clarification are still strongly needed.

We wish to thank the reviewer for the comments.

Major comments.

**Comment**

Page 4, line 17-22
After major revision, the authors have revised their manuscript by deleting the findings and its relevant descriptions concerning the sex and smoking status (such as Table 2 and Table 3 in the previous manuscript). Therefore, I think that these sentences should be deleted (or at least modified) in the revised manuscript. Instead, authors should add the descriptions about the “radiologic aggravation”, such as the results of the previous studies and unknown findings so far.

**Response**

As you mentioned, we deleted the descriptions concerning sex and smoking status and changed to the following on Page 4, Lines 16-23, “Other factors that are currently considered to aggravate pulmonary MAC disease are the presence of fibrocavitary type on radiography, a positive acid-fast bacilli (AFB) smear of sputum samples, and a larger affected area.^7,14,16-18^ However, there have been few studies of the factors that exacerbate pulmonary MAC disease without treatment, and they were generally judged based on the initiation of treatment as an indicator of aggravation. Thus, the aim of the present study was to clarify the significant predictors of radiological aggravations of pulmonary MAC disease using only the data of treatment-naïve patients.”

**Comment**
2. Page 3, line 10
Keywords should be modified. Namely, “sex” and “smoking status” should be removed from the keywords. Instead, authors might want to add the other keywords related to the radiological aggravation.
**Response**

We added “radiological aggravation” instead of “sex” and “smoking status” as a keyword.

**Comment**
3. Page 8, line 1, Page 8, line 7, Figure 1, Table 2
238 (the number of total patients with MAC lung disease) – 62 (the number of patients received treatment) = 176
However, in Figure 1 and Table 2, the number of treatment-naïve patients was 167. Please clarify this.

**Response**

We excluded 9 patients who could not be evaluated by chest CT one year after MAC diagnosis. Thus, we added the following on Page 5, Line 13 and the exclusion criteria of Figure 1 “radiographic findings were evaluated at the time of diagnosis and 1 year later.”

**Comment**
4. Discussion
1) The sentence and paragraph of Discussion section is too fragmentary (In particular, Page 10, line 5-line 14, Page 11, line 21-line 23). Authors should rewrite Discussion section in a more comprehensive way.

**Response**

As you mentioned, we changed the sentences, and rewrote the following on Page 9, line 19-Page 10, line 13, “The aim of the present study was to clarify the significant predictors of radiological aggravations of pulmonary MAC disease using only the data of treatment-naïve patients. To date, disease progression of pulmonary MAC disease was defined as either requiring the start of treatment^7,14,17^ or the presence of aggravation on radiological imaging^26,27^. In the present study, disease progression was defined as aggravation on radiological imaging. In some previous studies, the reason that the initiation of treatment was defined as an indicator of exacerbation was that MAC is indolent in nature, and thus, in many cases, radiological changes are difficult to evaluate on chest X-ray, detailed evaluation requires chest CT, and no radiological evaluation method for pulmonary MAC disease has been established globally. However, the timing of treatment may be biased by each doctor and each patient when using the initiation of treatment as evidence of exacerbation. For example, elderly patients tend to disagree with long-term medication, even if the doctor suspects deterioration and considers that they should be treated. Fortunately, in most of the present cases, CT was performed in our hospital, and it was possible to examine the changes in radiological evaluations. In the present study, radiological aggravation over one year was found in 56/167 (33.5%) of treatment-naïve subjects. Previous studies reported that about 20-40% and 50% of pulmonary MAC patients showed radiological aggravations after 5 and 10 years, respectively.^16,26^. In the present study, the frequency of radiological aggravations was relatively high within only one year because of the absence of treatment.”

**Comment**
2) In addition, I think that many descriptions in the Discussion section are generally irrelevant (In particular, Page 9 line 11-page 10 line 4, Page 10 line 12-line 14) to the main findings of the present study.

**Response**

As you mentioned, we think that page 9 line 11-page 10 line 4 is irrelevant to the main findings of the present study. We deleted page 10 lines 12-14, as mentioned. However, this is a summary statistic of MAC pulmonary disease, and in order to prove that there is no significant difference from the general pulmonary MAC disease reports, it has been shortened significantly.

**Comment**
3) Page 10, line 19-Page 11, line 4: These sentences are simple repetitions of those described in the Result section.

**Response**

As you mentioned, we deleted these sentences and changed to the following on Page 10, Line 19-Page 11, line12, “Previous studies reported that, in addition to extensive radiological findings, positive sputum AFB^7,17^ smear, FC type^7,16^, and lower BMI^16,18^ were aggravating factors. It is difficult to make a strict comparison between the current study and previous studies, because previous studies that treated patients are included, or they defined exacerbation as requiring treatment. In the present study, positive AFB smear, FC type, and lower BMI were not associated with radiological aggravations, but positive AFB smear (OR 3.020, 1.358-6.715) tended to be more common in patients with MAC disease aggravations.No studies examined the presence or absence of underlying lung diseases as an aggravating factor in pulmonary MAC disease. The present study demonstrated that the absence of other underlying lung diseases in untreated MAC patients was a significant aggravating factor of pulmonary MAC disease. Patients having other underlying lung diseases seemed to undergo radiological examinations more frequently than those without other underlying lung diseases. Thus, there may be more opportunities to identify the early phase of pulmonary MAC disease in those with underlying lung diseases. However, it cannot be ruled out that the diseases themselves, such as some kind of lung disease, and part of their treatment may be factors that suppress the progression of pulmonary MAC disease.^28^ These will be our future research targets.”

**Comment**

4) Page 11, line 4 “female sex, never-smoker”: In the revised manuscript, I think that there is no more reason to make an assumption that gender or smoking status are related to radiologic aggravation.

**Response**

Based on your comment, we deleted these sentences.

**Comment**

5) Page 11, line 9: these studies -> previous studies

**Response**

We replaced “these studies” with “previous studies”, as suggested.

**Comment**

6) Page 11, line 9-line 11: Please add reference for this sentence.

**Response**

We added a reference.

**Comment**
7) Page 11, line 12: the presence -> the presence or absence

**Response**

We replaced “the presence” with “the presence or absence”, as suggested.

**Comment**

8) Page 11, line 19-line 20: Please delete this sentence.

**Response**

We deleted this sentence, as suggested.

**Comment**

9) Page 12, line 2: limitations -> I think that the Authors’ scoring system has “merit” over the previous scoring system, rather than “limitation”.

**Response**

As you mentioned, we replaced “several limitations” with “some limitations and merits.”

**Comment**

10) Page 12, line 16-19: I do not believe that this is the limitation of the present study. Because the aim of the present study is to investigate the predictors of radiologic aggravation in “treatment-naïve” patients diagnosed with MAC lung disease “according to the ATS criteria”.

**Response**

Thank you for your comment. As suggested, we changed the limitations. “Some limitations of the present study should be addressed. This study was limited by its retrospective nature without randomization, and it was a single-institution study, and as such, it is not representative of the national population.Additionally, this was a short-term study, and the number of MAC patients may have been underestimated since patients who were not diagnosed according to the 2007 American Thoracic Society/Infectious Disease Society guideline were excluded from the analyses. Therefore, factors with clinical significance in reality may have proven insignificant in the analyses with reduced statistical power.”

**Comment**

11) Page 12, line 19-20 “the number of cases being treated was small”: Authors have already excluded the patients who received treatment. Therefore, these patients were not included in the main analysis of the present study.

**Response**

We deleted the sentences, and made the changes mentioned above.

**Comment**

12) Page 12, line 24 “the most critical factor”: As figure 2 shows, “no lung disease other than MAC” has higher OR than radiologic involvement. Therefore, I do not understand why “more extensive radiological findings” was the “most” critical factor of radiologic aggravation.

**Response**

Based on this comment, we deleted “most”.

**Comment**

Minor comments.

1. Page 2, line 14-15: the natural predictors of exacerbation -> the predictors of radiologic aggravation
**Response**

We corrected it as suggested.

**Comment**

2. Page 2, line 18: were common -> were predominant

**Response**

We changed it as suggested.

**Comment**

3. Page 5, line 13: also -> please delete
**Response**

We deleted it as suggested.

**Comment**

4. Page 7, line 3-line 6 -> please consider to delete this sentence

**Response**

We deleted this sentence as suggested.

**Comment**

5. Page 8, line 16-17, “whereas female sex ~ aggravations” -> please delete this sentence.

**Response**

We deleted this sentence as suggested.

**Comment**

6. Table 1, smoking history: 2+76+154+10 = 242, not 238

**Response**

We corrected it.

**Comment**

7. Table 1, previous tuberculosis: 23/238 = 9.7%, not 8.8%

**Response**

We corrected it.

**Comment**

8. Table 2: see footnotes of Table 1 and Table 2 -> see footnote of Table 1

**Response**

We corrected it.
