# Supplementary material for: Predictors of radiological aggravations of pulmonary MAC disease
Source: PLoS One. 2020 Aug 6;15(8):e0237071. doi: 10.1371/journal.pone.0237071 (PMC7410298; doi:10.1371/journal.pone.0237071)
Supplement: Attachment — Submitted filename: Reviewer comments.docx [file pone.0237071.s005.docx]

The authors have responded well to my suggestions and revised the paper superbly. However, some major corrections and further clarification are still strongly needed.

Major comments.

1. Page 4, line 17-22

After major revision, the authors have revised their manuscript by deleting the findings and its relevant descriptions concerning the sex and smoking status (such as Table 2 and Table 3 in the previous manuscript). Therefore, I think that these sentences should be deleted (or at least modified) in the revised manuscript. Instead, authors should add the descriptions about the “radiologic aggravation”, such as the results of the previous studies and unknown findings so far.

2. Page 3, line 10

Keywords should be modified. Namely, “sex” and “smoking status” should be removed from the keywords. Instead, authors might want to add the other keywords related to the radiological aggravation.

3. Page 8, line 1, Page 8, line 7, Figure 1, Table 2

238 (the number of total patients with MAC lung disease) – 62 (the number of patients received treatment) = 176

However, in Figure 1 and Table 2, the number of treatment-naïve patients was 167. Please clarify this.

4. Discussion

1) The sentence and paragraph of Discussion section is too fragmentary (In particular, Page 10, line 5-line 14, Page 11, line 21-line 23). Authors should rewrite Discussion section in a more comprehensive way.

2) In addition, I think that many descriptions in the Discussion section are generally irrelevant (In particular, Page 9 line 11-page 10 line 4, Page 10 line 12-line 14) to the main findings of the present study.

3) Page 10, line 19-Page 11, line 4: These sentences are simple repetitions of those described in the Result section.

4) Page 11, line 4 “female sex, never-smoker”: In the revised manuscript, I think that there is no more reason to make an assumption that gender or smoking status are related to radiologic aggravation.

5) Page 11, line 9: these studies 🡪 previous studies

6) Page 11, line 9-line 11: Please add reference for this sentence.

7) Page 11, line 12: the presence 🡪 the presence or absence

8) Page 11, line 19-line 20: Please delete this sentence.

9) Page 12, line 2: limitations 🡪 I think that the Authors’ scoring system has “merit” over the previous scoring system, rather than “limitation”.

10) Page 12, line 16-19: I do not believe that this is the limitation of the present study. Because the aim of the present study is to investigate the predictors of radiologic aggravation in “treatment-naïve” patients diagnosed with MAC lung disease “according to the ATS criteria”.

11) Page 12, line 19-20 “the number of cases being treated was small”: Authors have already excluded the patients who received treatment. Therefore, these patients were not included in the main analysis of the present study.

12) Page 12, line 24 “the most critical factor”: As figure 2 shows, “no lung disease other than MAC” has higher OR than radiologic involvement. Therefore, I do not understand why “more extensive radiological findings” was the “most” critical factor of radiologic aggravation.

Minor comments.

1. Page 2, line 14-15: the natural predictors of exacerbation 🡪 the predictors of radiologic aggravation

2. Page 2, line 18: were common 🡪 were predominant

3. Page 5, line 13: also 🡪 please delete

4. Page 7, line 3-line 6 🡪 please consider to delete this sentence

5. Page 8, line 16-17, “whereas female sex ~ aggravations” 🡪 please delete this sentence.

6. Table 1, smoking history: 2+76+154+10 = 242, not 238

7. Table 1, previous tuberculosis: 23/238 = 9.7%, not 8.8%

8. Table 2: see footnotes of Table 1 and Table 2 🡪 see footnote of Table 1
